# Clinicopathological Spectrum of Hodgkin's and Non-Hodgkin's Lymphoma: A Tertiary Care Cancer Hospital Study in Pakistan

**DOI:** 10.7759/cureus.25620

**Published:** 2022-06-03

**Authors:** Wardah Aslam, Maryam Habib, Saeeda Aziz

**Affiliations:** 1 Pathology/Hematology, Nuclear Medicine, Oncology and Radiotherapy Institute, Islamabad, PAK; 2 Pathology/Hematology, Shifa College of Medicine (STMU), Islamabad, PAK; 3 Pathology/Clinical Pathology, Nuclear Medicine, Oncology and Radiotherapy Institute, Islamabad, PAK

**Keywords:** neoplasms, hodgkin's disease, non-hodgkin lymphoma, t-cell, b-cell, lymphoma

## Abstract

Introduction

Lymphomas are a heterogeneous group of disorders that arise primarily from lymphoid tissue and are categorized based on histological features and immunophenotypes. The distribution and frequency of different types of lymphoma vary in different parts of the world. This study aimed to document the frequency and clinicopathological characteristics of various types of lymphoma in our population to understand the ever-increasing burden of disease and formulate the optimal management and prevention plans.

Materials and methods

This study was conducted at Nuclear Medicine, Oncology and Radiotherapy Institute (NORI) from August 2015 to March 2022. A total of 300 cases of lymphoma that were diagnosed and treated at NORI were included in the study. We measured the frequency of different lymphomas and patient age, sex, and stage IV presentation at the time of diagnosis. IBM SPSS Statistics for Windows, Version 23.0 (Armonk, NY: IBM Corp.) was used to analyze the data.

Results

Three hundred patients with lymphoma were included in the study. There were more non-Hodgkin’s lymphoma (NHL) cases (n=224; 74.6%) than Hodgkin’s lymphoma (HL) cases (n=76; 25.3%). T-cell NHL was seen in 11 cases (4.8%), while B-cell NHL was found in 214 cases (95%). Diffuse large B-cell lymphoma was the predominant type (n=156; 69.3%). Among T-cell lymphomas, anaplastic T-cell lymphoma was the most common subtype (n=6; 2.6%) followed by angioimmunoblastic T-cell lymphoma (n=2; 0.8%) and T-cell lymphoblastic lymphoma (n=1; 0.4%). For classical HL, mixed cellularity was the predominant type (n=38; 50%) followed by nodular sclerosis (n=31; 40.8%), lymphocyte depleted (n=5; 6.6%), and lymphocyte rich (n=2; 2.6%). Stage IV was present in 21 HL cases (27.6%), and stage IV was seen in 67 NHL cases (29.7%) at the time of diagnosis. Most HL and NHL patients were male. Most HL cases presented in the younger age group (aged 15 to 35 years), while the largest group of NHL patients were aged 56 to 75 years.

Conclusion

Our population has a broad spectrum of lymphoma and its subtypes. NHL is more common than HL, and the frequency of B-cell NHL is higher than that of T-cell NHL. Approximately one-third of the patients presented in stage IV at the time of diagnosis. An awareness of clinicopathological characteristics of lymphoma in our setup would aid in diagnosis, formulating standard management plans, and prevention strategies for optimal patient outcomes.

## Introduction

Lymphomas are a diverse group of neoplasms that develop from lymphocytes and other organs involved in the lymphatic system. Lymphomas are broadly categorized into Hodgkin's lymphoma (HL) and Non-Hodgkin's lymphoma (NHL). NHLs are a heterogeneous group of neoplastic diseases. They are broadly divided into B, T, and natural killer (NK) cell lymphomas. Based on World Health Organization (WHO) classification, they are categorized into different types. Lymphomas can be nodal or extranodal, depending upon their primary site of involvement. HL is further subdivided into the classical type and nodular lymphocyte-predominant type. Classical HL is further subdivided into five types [1,2]. Lymphomas are mostly diagnosed based on the biopsy of the involved lymphoid tissue and staged according to the Ann Arbor system (I-IV), depending on the spread of the disease [3].

The incidence of cancer has increased by almost 30% worldwide in the last few decades. Global cancer statistics show that the burden of NHL has also increased, accounting for approximately 4% to 6% of total cancers. NHL is the eighth leading cause of cancer death in the United States [4-6]. Lymphoma is also a significant part of pediatric cancers. To avoid unnecessary delays in diagnosis, lymphoma is diagnosed through clinicopathological teleconferences in some lymphoma-endemic regions [7]. Various studies have found different etiologic factors behind the increased number of lymphoma cases. Some of the factors that have been identified as influencing cancer incidence, especially lymphoma, include a polluted environment containing a wide variety of known human carcinogens. Similarly, soil contaminated with chemicals and carcinogens can also be a risk factor for malignancy [8,9]. Other studies have also emphasized the effect of poverty, infection, and human immunodeficiency virus/acquired immunodeficiency syndrome on the growing cancer trends worldwide [10].

Like in other parts of the world, the lymphoma burden has increased in Pakistan over the past few decades. There are different regional registries in Pakistan, but no central registry is available [11]. Given the increase in lymphoma cases and the scarcity of data in this regard, a comprehensive study about the lymphoma burden in Pakistan is an urgent necessity. The present study aimed to find the frequency of different types of lymphoma in our institute, along with their associated clinical manifestations and stage IV presentation at the time of diagnosis. A preliminary abstract of this study was presented at the World Congress of the International Society of Hematology in 2018 (Faheem M, Aslam W, Habib M, Mehmood H, Zafar S. A Large Single Centre Study on Frequency and Stage IV Presentation of Hodgkin and Non-Hodgkin Lymphoma in Pakistan. XXXVII World Congress of International Society of Hematology; 2018).

## Materials and methods

We conducted this cross-sectional study at the Nuclear Medicine, Oncology and Radiotherapy Institute (NORI) from August 2015 to March 2022. The study protocol was approved by the Ethics Committee and Institutional Review Board of NORI (IRB No. 2(10)/88). The study included all patients diagnosed at this hospital after complete history, examination, and histopathological and hematological analysis and had no secondary malignancies. The study excluded all patients with a secondary disease or incomplete clinical and laboratory data.

NORI is one of the largest government hospitals in Islamabad, the capital of Pakistan. NORI is equipped with all modern diagnostic facilities and treatment options and contains a team of experienced and well-qualified hematologists, histopathologists, radiologists, oncologists, and nuclear medicine experts. Being a government sector hospital, the charges for diagnostic and therapeutic facilities are minimal compared to private sector hospitals. Patients are referred from all over the country to this hospital. Given its location, its patient population is an accurate representation of patients from northern Pakistan and Pakistan's tribal regions. Most patients attending this hospital are low-income, poor socioeconomic status.

Histopathological diagnosis and staging

All the data were retrieved from patient case files and the laboratory information system. During the approximately seven-year study, 300 patients were diagnosed with lymphoma and treated at this hospital. We collected standard history and performed complete examinations on all patients. We noted the presence or absence of B symptoms. Patients were staged entirely after magnetic resonance imaging and a computed tomography scan of the neck, chest, abdomen, and pelvis. All patients received bone marrow examinations and reports by hematologists. We assessed blood chemistry for all patients and conducted a complete blood count with differential leukocyte count and peripheral smear examination. All the biopsies taken from patients were processed and analyzed by histopathologists, and immunohistochemical staining was applied for morphological diagnosis of biopsies. Cytogenetic analysis was not available in most cases. A final diagnosis and categorization were made according to 2016 WHO classification [1].

Data analysis

All the data were noted and analyzed using IBM SPSS Statistics for Windows, Version 23.0 (Armonk, NY: IBM Corp.). We calculated frequency and percentages for qualitative variables. We obtained the mean and standard deviation of quantitative variables.

## Results

**Figure 1:**
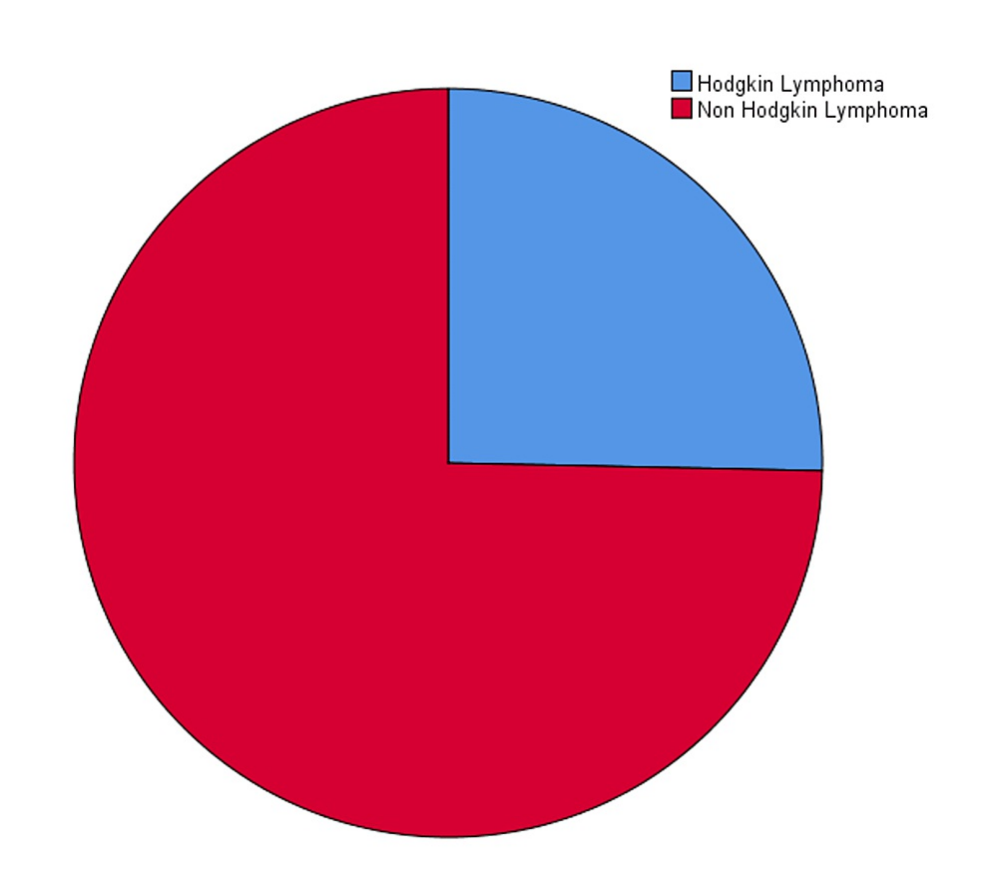
Types of lymphoma

**Figure 2:**
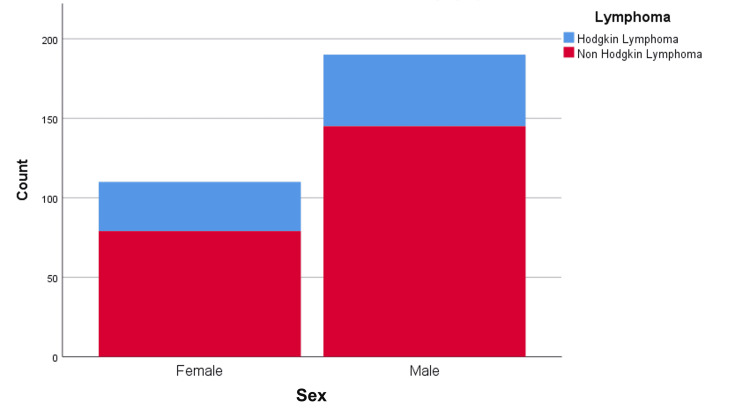
Gender distribution in lymphoma patients

The study population consisted of 300 patients ranging in age from 15 to 85 years. The median age of patients was 56 ± 20.22 years. The study included 190 male and 110 female patients (male-to-female ratio: 1.72:1.79). Most patients (n=224; 74.67%) were diagnosed with NHL, while 76 patients (25.33%) were diagnosed with HL (Figure 1). One hundred forty-five male patients (48.33%) and 79 (26.33%) female patients were diagnosed with NHL. Forty-five male patients (15%) and 31 female patients (10.33%) were diagnosed with HL (Figure 2).

**Figure 3:**
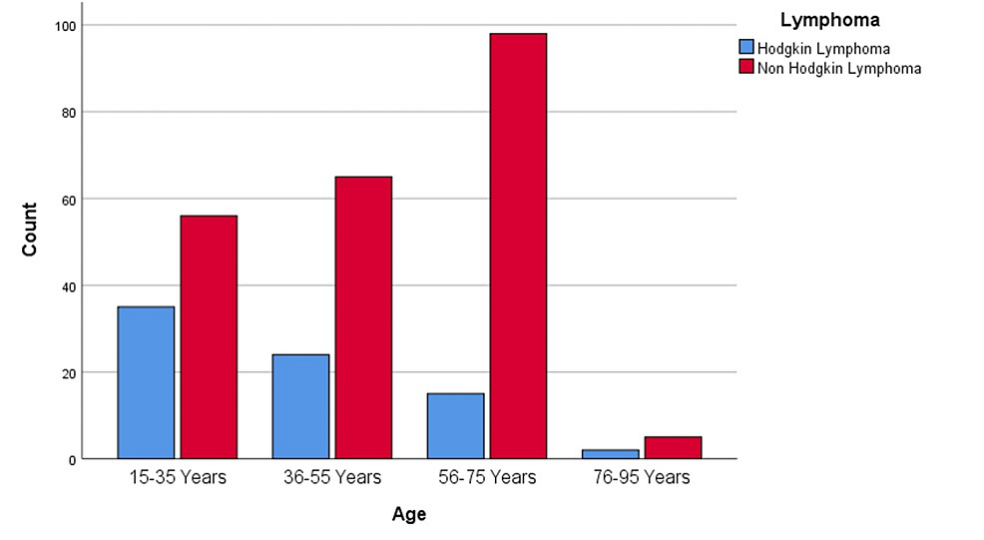
Age statistics in lymphoma patients

Most patients with HL were aged 15 to 35 years (n=35; 11.67%), and the incidence of HL decreased steadily across the older age groups (Figure 3). By contrast, NHL was more prevalent among patients of advanced age: the 56- to 75-year age group had 98 patients (32.67%), the 36- to 55-year age group had 65 patients (21.67%), and the 15- to 35-year age group had 56 patients (18.67%). The oldest age group (76 to 95 years) had only five NHL patients (1.67%).

**Figure 4:**
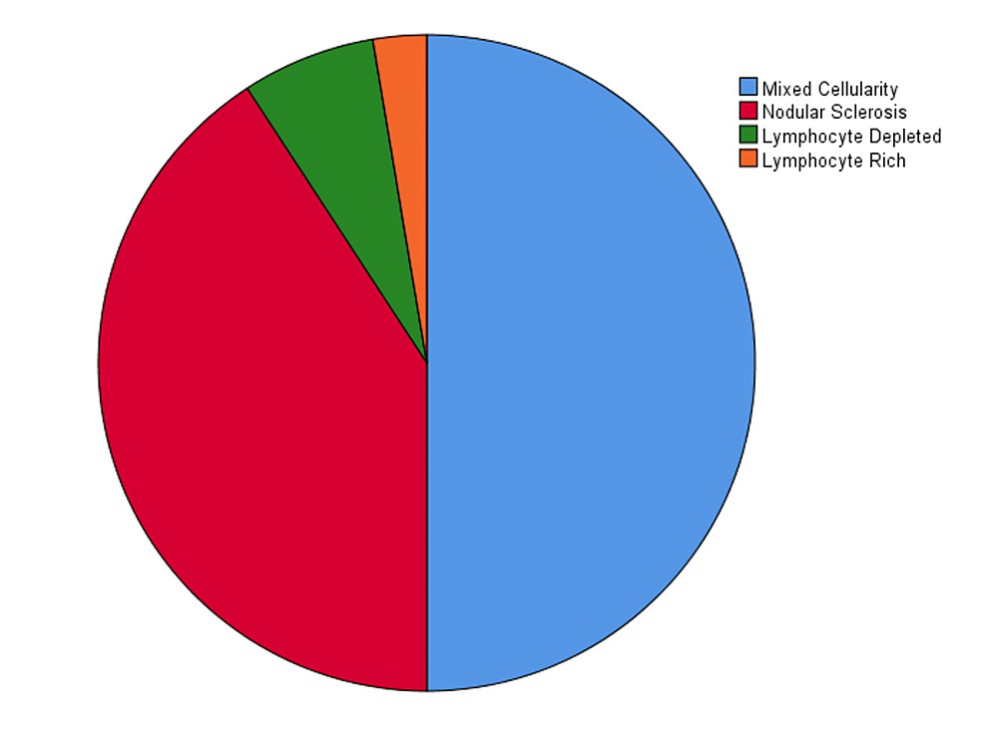
Frequency of subtypes of Hodgkin's lymphoma

**Figure 5:**
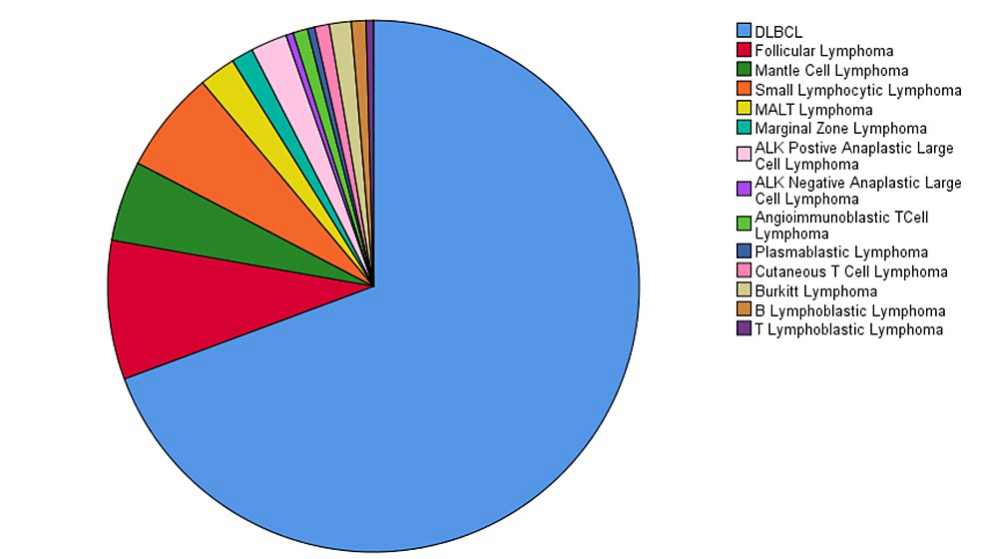
Distribution of non-Hodgkin's lymphoma DLBCL, diffuse large B-cell lymphoma; MALT, mucosa-associated lymphoid tissue; ALK, anaplastic lymphoma kinase positive.

We classified HL into subtypes and found that mixed cellularity was the most common type (n=38; 50%) followed by nodular sclerosis (n=31; 40.79%), lymphocyte depleted (n=5; 6.58%), and lymphocyte rich (n=2; 2.63%) in decreasing order of frequency (Figure 4). NHL was categorized into B- and T-cell lymphomas which were further subtyped. Among B-cell NHL, diffuse large B-cell lymphoma (DLBCL) was the most common type (n=156; 69.3%) followed by follicular lymphoma (n=19; 8.4%), small cell lymphoma (n=14; 6.2%), mantle cell lymphoma (n=11; 4.9%), mucosa-associated lymphoid tissue lymphoma (n=5; 2.2%), marginal zone lymphoma (n=3; 1.3%), Burkitt's lymphoma (n=3; 1.3%), B-cell acute lymphoblastic lymphoma (n=2; 0.9%), and plasmablastic lymphoma (n=1; 0.4%; Figure 5).

Eleven patients (4.8%) were diagnosed with T-cell NHL. The most common (n=6; 2.6%) was anaplastic large cell NHL. We further classified these based on anaplastic lymphoma kinase (ALK) positivity. Of six anaplastic large cell lymphoma patients, five were ALK positive, and we saw only one case of ALK-negative anaplastic large cell lymphoma. We had two patients (0.8%) with cutaneous T-cell lymphoma and angioimmunoblastic lymphoma, while only one patient was diagnosed with T-cell acute lymphoblastic lymphoma (0.4%). We also recorded the incidence of stage IV lymphomas in our patients (Tables 1, 2). Of 76 HL cases, we noted stage IV in 21 patients. Of the 225 NHL cases, 67 patients had stage IV at presentation.

**Table 1:** Prevalence of stage IV disease in Hodgkin’s lymphoma

	Stage IV			
	Absent		Present	
Hodgkin lymphoma	N	Percentage	N	Percentage
Mixed cellularity	26	47.3%	12	57.1%
Nodular sclerosis	24	43.6%	7	33.3%
Lymphocyte depleted	3	5.5%	2	9.5%
Lymphocyte rich	2	3.6%	0	0.0%

**Table 2:** Prevalence of stage IV disease in non-Hodgkin’s lymphoma DLBCL, diffuse large B-cell lymphoma; MALT, mucosa-associated lymphoid tissue; ALK, anaplastic lymphoma kinase positive.

	Stage IV			
	Absent		Present	
Non-Hodgkin lymphoma	N	Percentage	N	Percentage
DLBCL	110	69.6%	46	68.7%
Follicular lymphoma	13	8.2%	6	9.0%
Mantle cell lymphoma	8	5.1%	3	4.5%
Small lymphocytic lymphoma	8	5.1%	6	9.0%
MALT lymphoma	3	1.9%	2	3.0%
Marginal zone lymphoma	3	1.9%	0	0.0%
ALK-positive anaplastic large cell lymphoma	4	2.5%	1	1.5%
ALK-negative anaplastic large cell lymphoma	1	0.6%	0	0.0%
Angioimmunoblastic T-cell lymphoma	2	1.3%	0	0.0%
Plasmablastic lymphoma	1	0.6%	0	0.0%
Cutaneous T-cell lymphoma	1	0.6%	1	1.5%
Burkitt's lymphoma	2	1.3%	1	1.5%
B lymphoblastic lymphoma	1	0.6%	1	1.5%
T lymphoblastic lymphoma	1	0.6%	0	0.0%

## Discussion

This study's goal was to report the frequency of various types of lymphoma in our institution. Pakistan is included in the so-called "lymphoma belt," stretching from Southeastern Asia, across the Middle East and Northern Africa. There has been an alarming increase in lymphoma cases in the past few decades in our region and country [12,13]. While many studies have been conducted in the last decade on the incidence and prevalence of lymphoma in Pakistan, no recent study has been conducted on the ever-increasing burden of lymphoma in Pakistan.

We reported 300 cases of lymphoma, which were further subtyped into HL and NHL. NHL cases were further divided into B- and T-cell lymphomas. In concordance with studies conducted worldwide, B-cell lymphomas were the predominant type of NHL in our population. Our results were similar to findings reported by Jiang et al., who emphasized that T-cell lymphomas constitute only 10% to 15% of NHL cases [14]. Of B-cell NHL cases, DLBCL was the most common subtype in our cohort. These results are similar to the studies conducted by Pervez, Susanibar-Adaniya and Barta, and Wang et al. [15-17], which showed that DLBCL was the most common type of NHL. Follicular lymphoma was the second most predominant lymphoma in our study. However, Pervez showed a lower prevalence of follicular lymphoma in their study [18]. They suggested that a lack of early diagnosis and proper diagnostic modalities may be the reason behind the smaller incidence of other types of NHL apart from DLBCL. The recent advent of more advanced facilities in Pakistan has mitigated these issues [18]. DLBCL was the most common type among our NHL patients, followed by follicular, small lymphocytic, and mantle cell lymphoma. The study on NHL by Pervez in Pakistan found that small lymphocytic lymphoma was second most common after DLBCL, followed by follicular and mantle cell lymphoma [15]. In our study, the most common type of T-cell NHL was anaplastic large cell lymphoma, aligned with the findings of Pervez [15].

Mixed cellularity was the most common type of HL, followed by nodular sclerosis, lymphocyte depleted, and lymphocyte-predominant HL. A large-scale study of 650 patients with HL by Siddiqui et al. and a study of 36 patients with HL by Nawaz et al. also reported that mixed cellularity was the most common subtype of HL with a male predominance [19,20]. Compared to other regions, the incidence and distribution of lymphoma cases in our part of the world revealed certain similarities and specific differences. A study of lymphoma by Polepole et al. in a Zambian population showed that the prevalence of NHL and HL in their country was somewhat similar to our findings [21]. Similar results were reported by Shanbhag and Ambinder, who reported Burkitt's lymphoma and DLBCL occurred in equal proportions in their population, while the incidence of Burkitt's lymphoma was much lower than that in DLBCL in our population [22]. Samiee et al. conducted a study in Iran that showed DLBCL was the most common B-cell NHL, which was similar to our study; however, among their T-cell NHL cases, large granular lymphocytic lymphoma and anaplastic large cell lymphoma were the most common subtypes while we had predominantly anaplastic large cell lymphoma cases [23].

With the increase in lymphoma cases worldwide, especially in South East Asia, it is important to know the local prevalence of lymphomas and their demographics, which can help generate optimal management plans. NHL was more common than HL in Pakistan and other areas of the world; however, the incidence and frequency of different subtypes of lymphomas differ locally from Western countries [24-26]. We found a male predominance in HL and NHL, similar to results from other studies [27,28]. Our age statistics were similar to other studies, with most cases occurring among 56- to 75-year-olds. However, a study conducted by Sharma et al. revealed a bimodal peak of lymphoma in their patients, which was not present in our population [29].

With better diagnostic facilities, new lymphoma cases are being diagnosed and classified promptly. Approximately one-third of our patients presented in stage IV. This staging might be linked to their poor socioeconomic status and difficulties in seeking healthcare from far northern areas of the country. The difference in the prevalence of lymphoma subtypes in Western countries might be due to the variations in the population's genetic makeup, the effect of different viruses, and environmental agents.

Our study has several significant limitations. The study does not represent the absolute number of lymphoma cases throughout Pakistan due to the lack of a central registry. More studies need to be conducted on a larger scale focusing on the exact number of lymphoma cases throughout Pakistan and identifying different etiological agents responsible for the ever-increasing numbers of lymphoma cases in Pakistan.

## Conclusions

Lymphoma presents across a spectrum of conditions in different parts of the world, and it is increasing in incidence in our region. The present study shows the prevalence of different kinds of HL and NHL in Pakistan. We categorized the frequency of different types of classical HL and identified the frequency of different subtypes of B-cell and T-cell NHL. NHL is more common than HL, and the frequency of B-cell NHL is higher than that of T-cell NHL. Approximately one-third of patients presented in stage IV at the time of diagnosis. A thorough understanding of the clinicopathological characteristics of lymphoma in our area would aid in diagnosis, formulating standard management plans and prevention strategies for optimal patient outcomes.
